# Supplementary figures and images for: Serine palmitoyltransferase assembles at ER–mitochondria contact sites
Source: Life Sci Alliance. 2021 Nov 16;5(2):e202101278. doi: 10.26508/lsa.202101278 (PMC8605320; doi:10.26508/lsa.202101278)

Source data - Figure 1

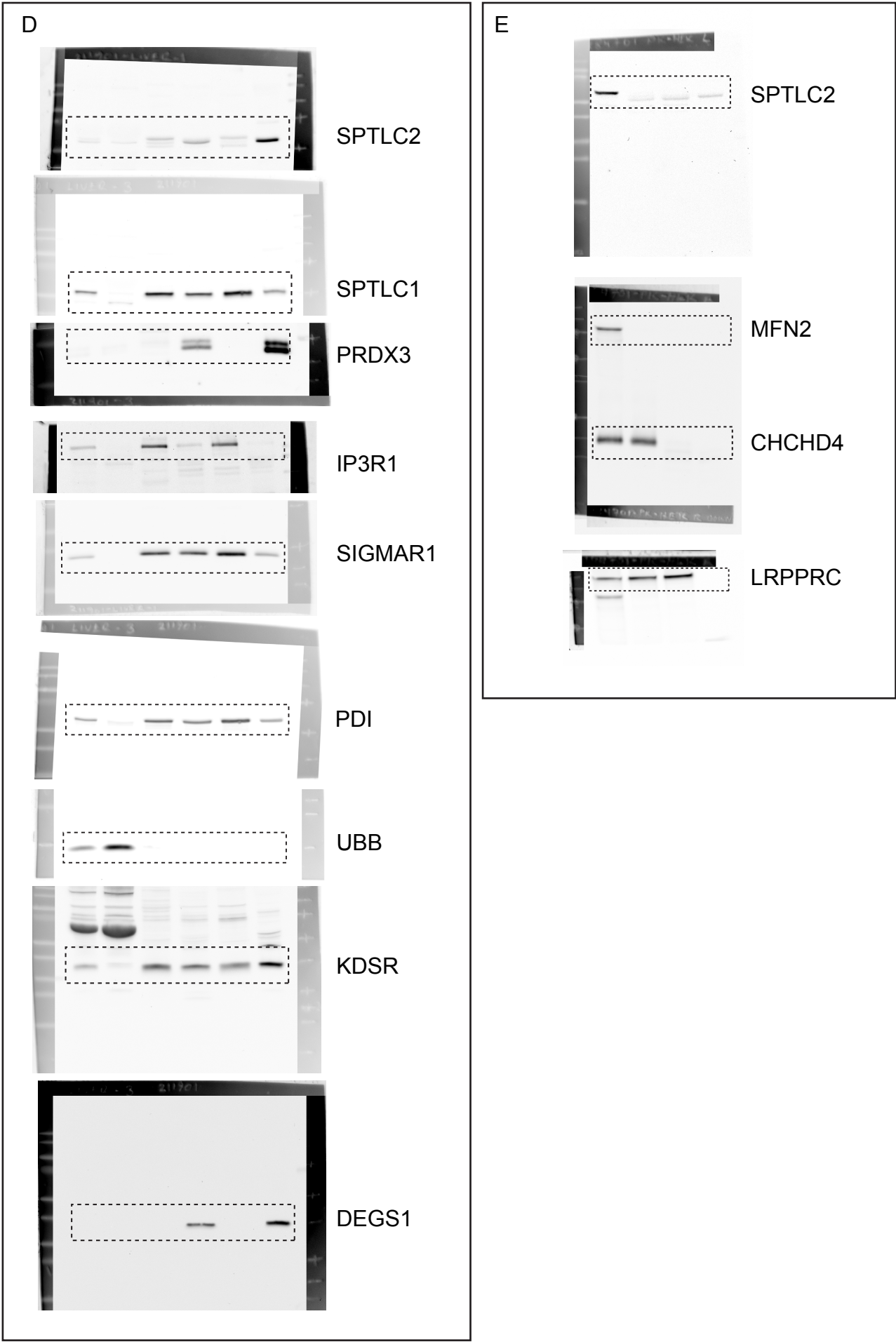

Supplement: Supplementary file 1 [file LSA-2021-01278_SdataF1.pdf]

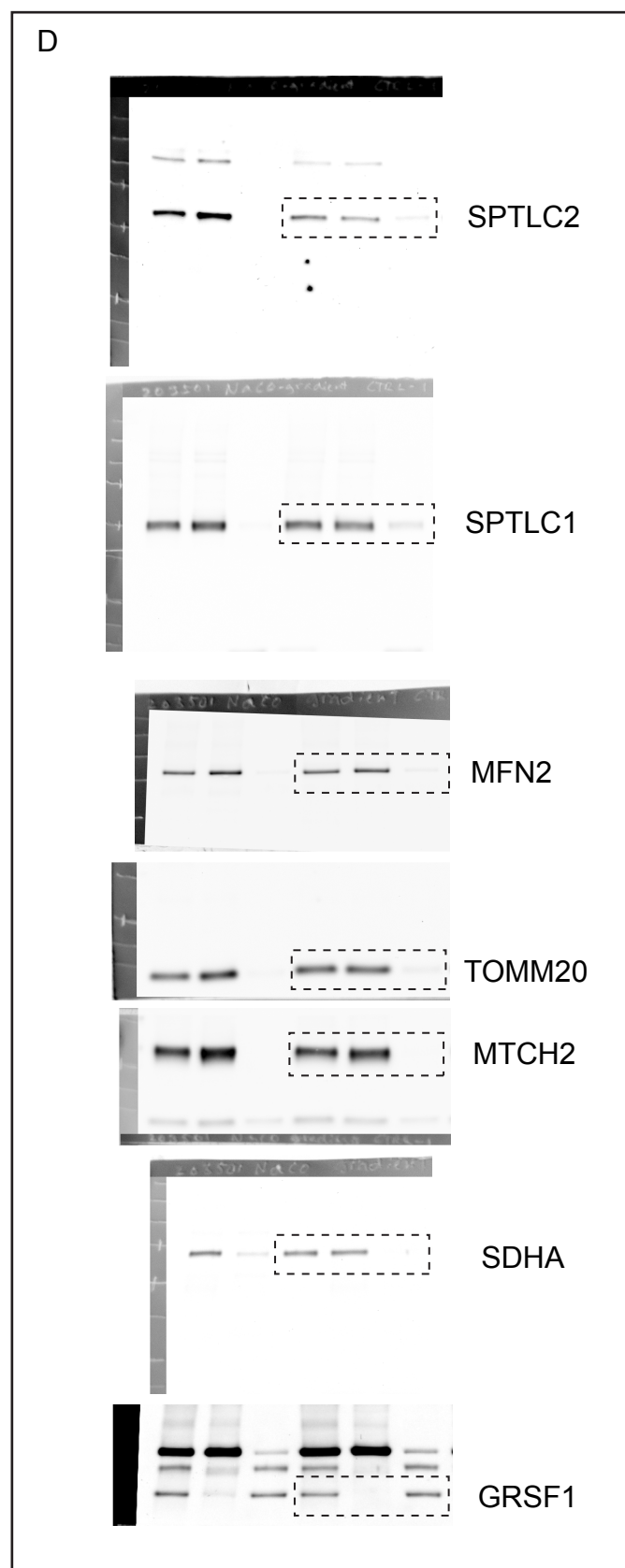

Source Data - Figure EV2

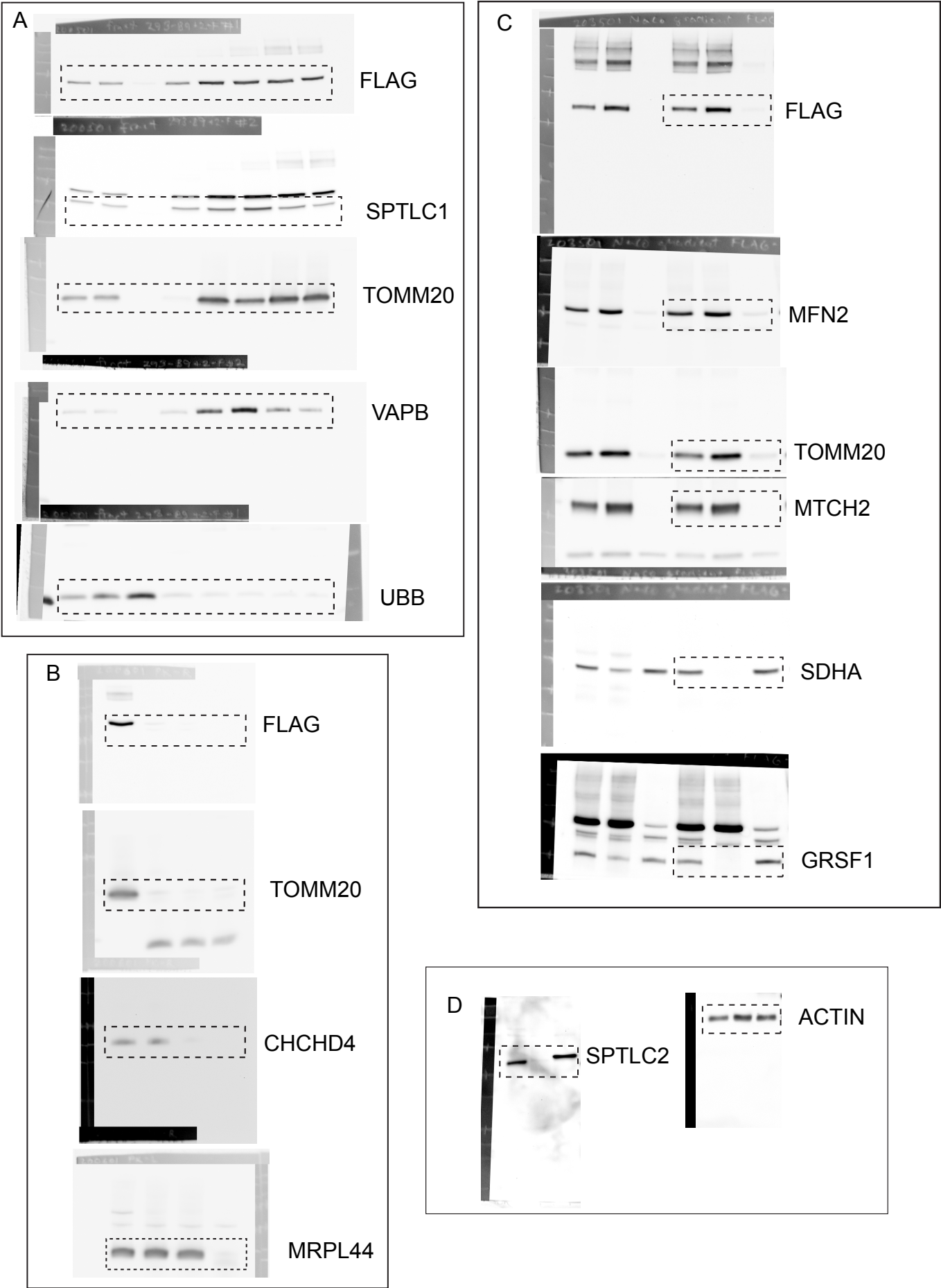

Source Data - Figure EV3

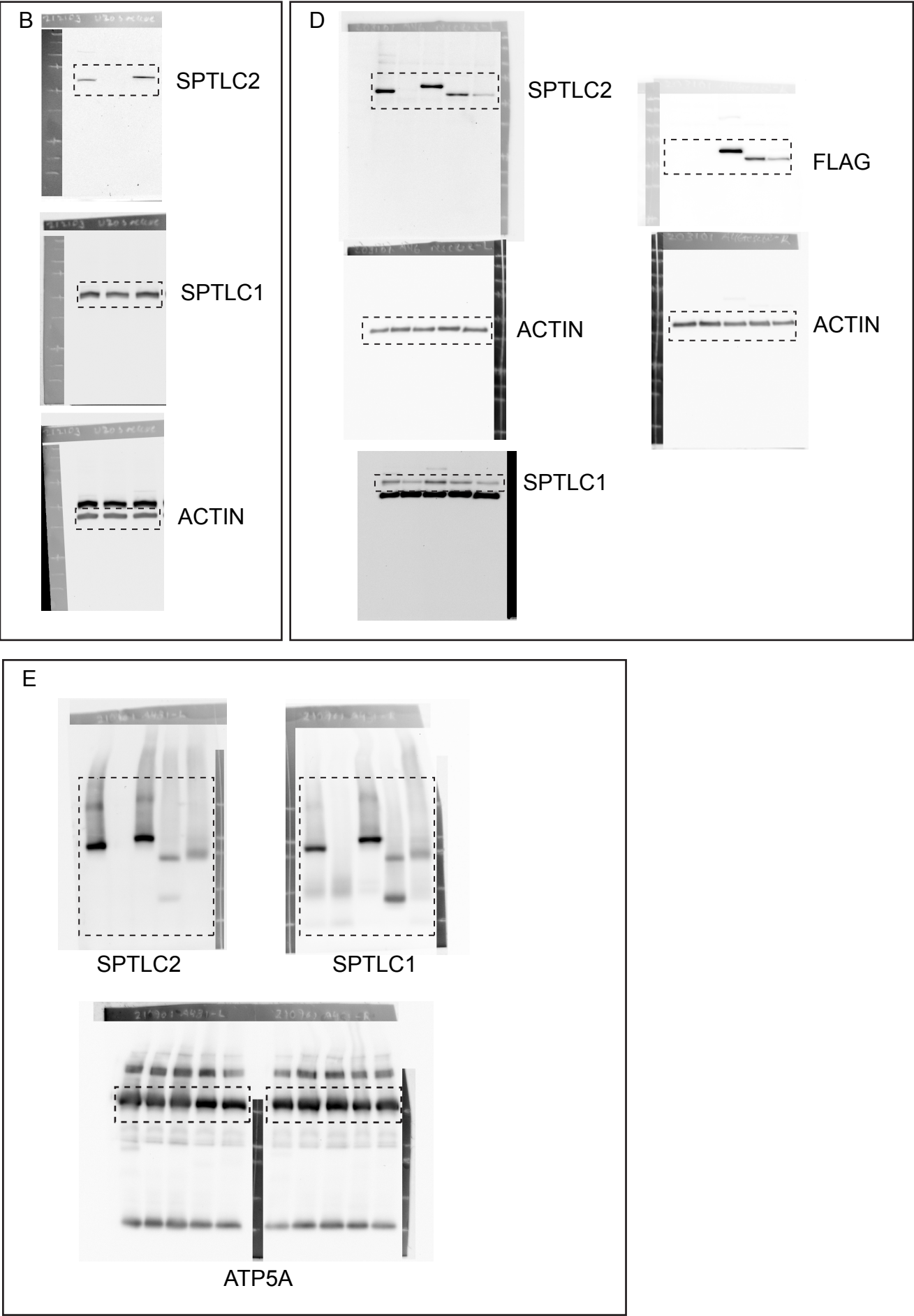

Source Data - Figure EV4

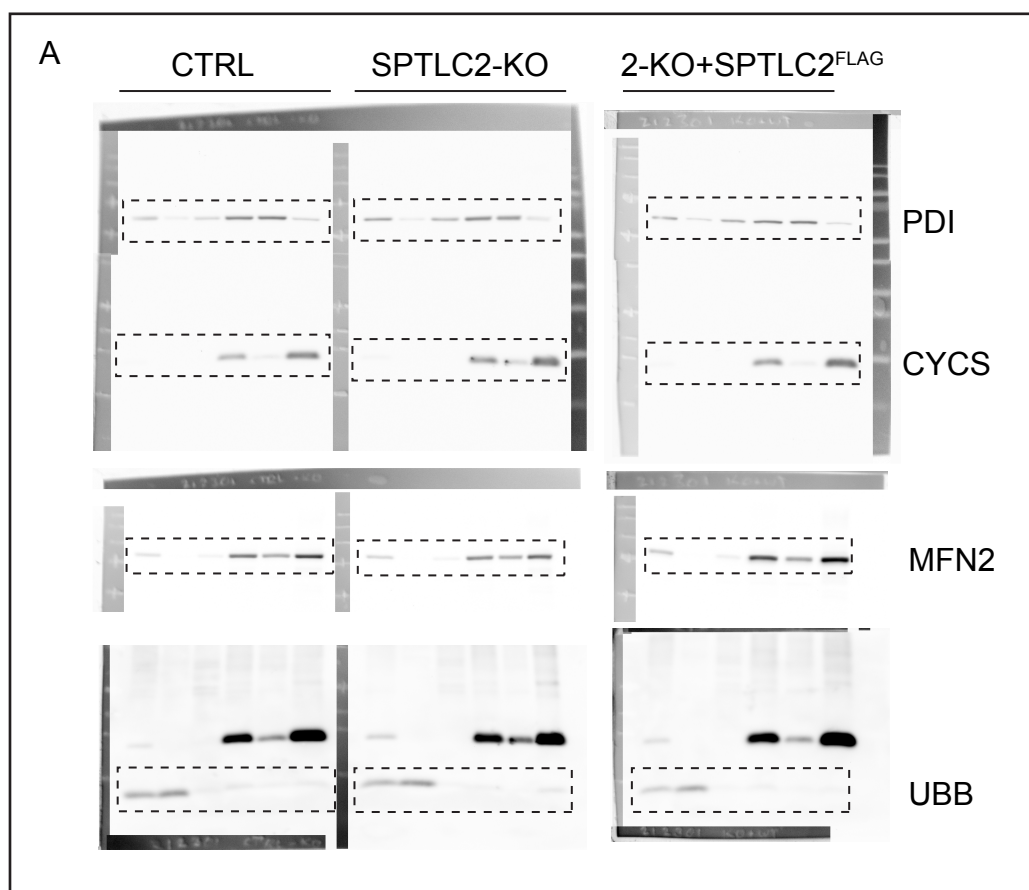

Supplement: Supplementary file 2 [file LSA-2021-01278_SdataFS1.S2.S3.S4.pdf]

Source Data - Figure 2

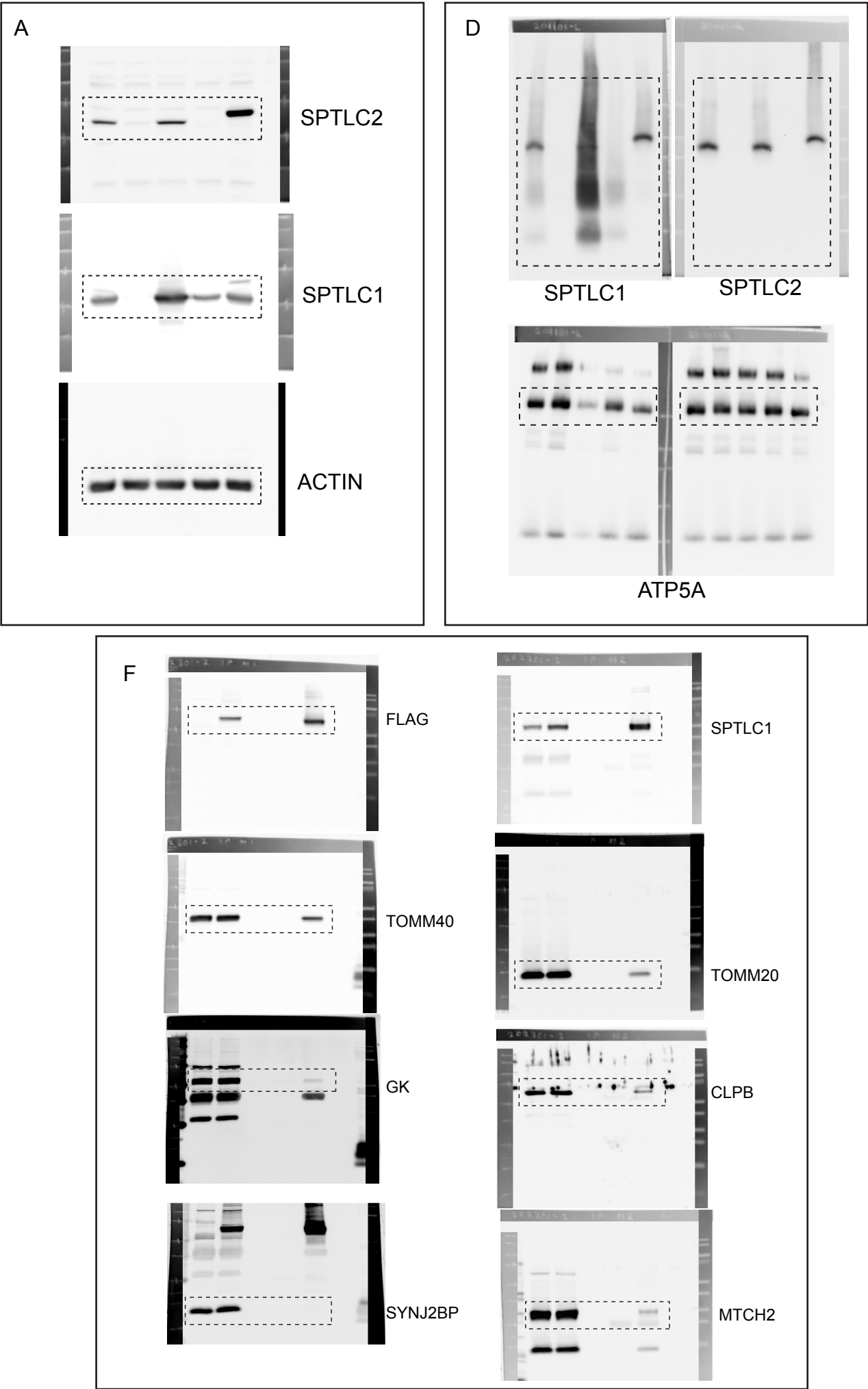

Supplement: Supplementary file 3 [file LSA-2021-01278_SdataF2.1.pdf]

Source Data - Figure 3

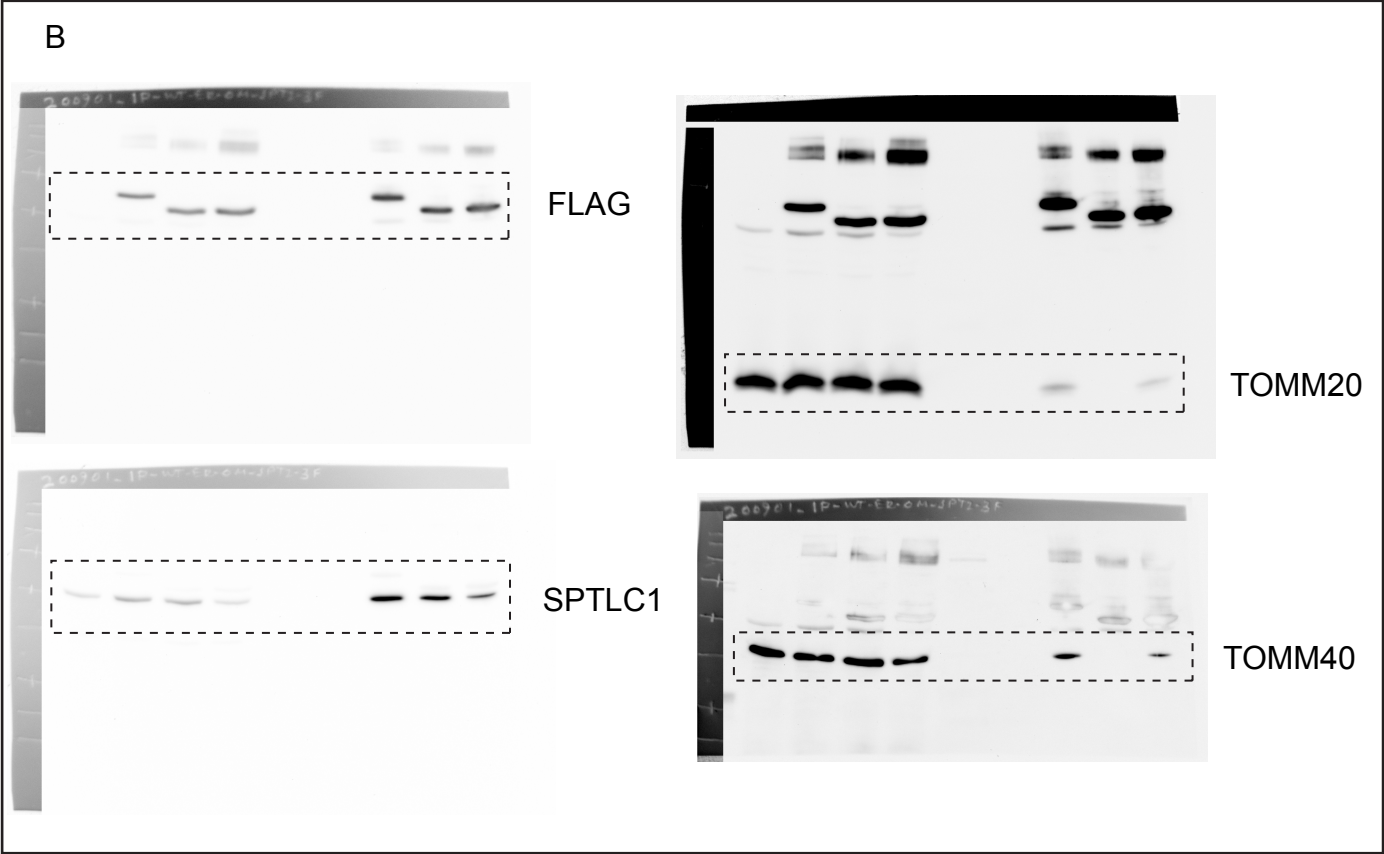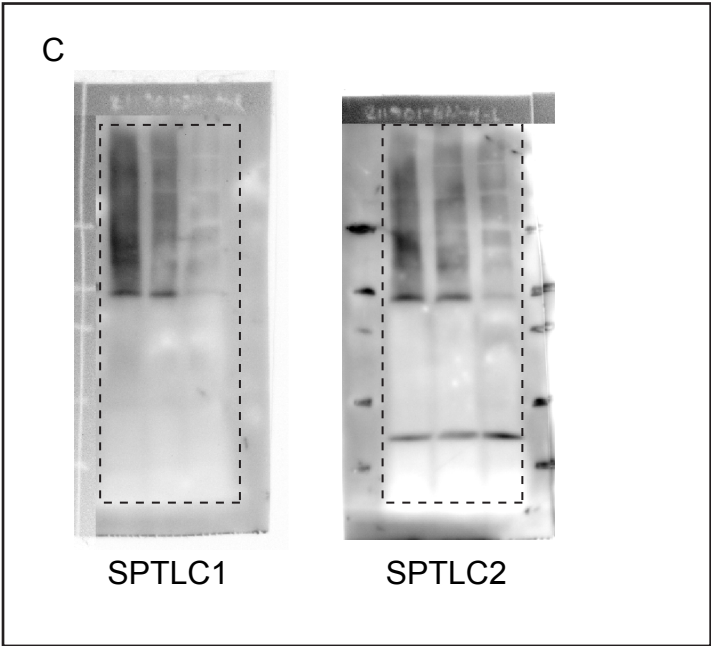

Supplement: Supplementary file 5 [file LSA-2021-01278_SdataF3.pdf]
